# Protective and Pain-Killer Effects of AMC3, a Novel N-Formyl Peptide Receptors (FPRs) Modulator, in Experimental Models of Rheumatoid Arthritis

**DOI:** 10.3390/antiox12061207

**Published:** 2023-06-02

**Authors:** Valentina Ferrara, Alessandra Toti, Elena Lucarini, Carmen Parisio, Laura Micheli, Clara Ciampi, Francesco Margiotta, Letizia Crocetti, Claudia Vergelli, Maria Paola Giovannoni, Lorenzo Di Cesare Mannelli, Carla Ghelardini

**Affiliations:** 1 Department of Neuroscience, Psychology, Drug Research and Child Health—NEUROFARBA—Pharmacology and Toxicology Section, University of Florence, 50139 Florence, Italy; valentina.ferrara@unifi.it (V.F.); alessandra.toti@unifi.it (A.T.); carmen.parisio@unifi.it (C.P.); laura.micheli@unifi.it (L.M.); clara.ciampi@unifi.it (C.C.); francesco.margiotta@unifi.it (F.M.); lorenzo.mannelli@unifi.it (L.D.C.M.); carla.ghelardini@unifi.it (C.G.); 2 Department of Neuroscience, Psychology, Drug Research and Child Health—NEUROFARBA—Pharmaceutical and Nutraceutical Section, University of Florence, 50139 Florence, Italy; letizia.crocetti@unifi.it (L.C.); claudia.vergelli@unifi.it (C.V.); mariapaola.giovannoni@unifi.it (M.P.G.)

**Keywords:** rheumatoid arthritis, pain, cartilage, chondrocyte, interleukin-1β, inflammation, oxidative stress, VEGF-A, disease modifying agent, pharmacological modulator

## Abstract

Rheumatoid arthritis is an autoimmune disorder that causes chronic joint pain, swelling, and movement impairment, resulting from prolonged inflammation-induced cartilage and bone degradation. The pathogenesis of RA, which is still unclear, makes diagnosis and treatment difficult and calls for new therapeutic strategies to cure the disease. Recent research has identified FPRs as a promising druggable target, with AMC3, a novel agonist, showing preclinical efficacy in vitro and in vivo. In vitro, AMC3 (1–30 µM) exhibited significant antioxidant effects in IL-1β (10 ng/mL)-treated chondrocytes for 24 h. AMC3 displayed a protective effect by downregulating the mRNA expression of several pro-inflammatory and pro-algic genes (iNOS, COX-2, and VEGF-A), while upregulating genes essential for structural integrity (MMP-13, ADAMTS-4, and COLIAI). In vivo, AMC3 (10 mg kg^−1^) prevented hypersensitivity and restored postural balance in CFA-injected rats after 14 days. AMC3 attenuated joint alterations, reduced joint inflammatory infiltrate, pannus formation, and cartilage erosion. Chronic AMC3 administration reduced transcriptional changes of genes causing excitotoxicity and pain (EAATs and CCL2) and prevented morphological changes in astrocytes, including cell body hypertrophy, processes length, and thickness, caused by CFA in the spinal cord. This study demonstrates the usefulness of AMC3 and establishes the groundwork for further research.

## 1. Introduction

Rheumatoid arthritis (RA) is a chronic autoimmune disorder that causes joint inflammation, pain, and stiffness. The onset of RA is influenced by a complex interplay of genetic, environmental, and lifestyle factors [1]. The joints are the primary site of inflammation in RA, which can cause significant morbidity and disability [2]. Pain is a predominant symptom arising from peripheral and central sensitization mechanisms [3]. Current therapeutic strategies include non-steroidal anti-inflammatory drugs (NSAIDs), glucocorticoids (GCs), and disease-modifying anti-rheumatic drugs (DMARDs), but these drugs promote side effects and are expensive [4]. To overcome these challenges, researchers are developing new molecules to improve efficacy and safety profiles [5].

RA pathogenesis involves the interaction among macrophages, fibroblast-like synoviocytes (FLS) [6], and chondrocytes [7]. This process is marked by the excessive production of inflammatory cytokines, resulting in synoviocyte hyperplasia and the formation of locally invasive synovial tissue, known as “pannus” [8,9]. In normal conditions, chondrocytes play a critical role in maintaining extracellular matrix (ECM) organization and composition of articular cartilage [10] through the secretion of proteases, such as matrix metallopeptidases (MMPs) and a disintegrin and metalloproteinase with thrombospondin motifs (ADAMTs) [11]. However, in joint diseases like RA, pro-inflammatory cytokines released by the synovium, including Interleukin (IL)-1β, -6, -17, and Tumor Necrosis Factor (TNF)-α, contribute to chondrocyte death [10] and matrix disruption [12,13]. Additionally, these cytokines promote the production of growth factors that activate non-neuronal cells, leading to the establishment of peripheral and central sensitization [14,15].

Inflammation resolution was once thought to be a passive process, but recent research has revealed it to be an active response mediated by specific endogenous pro-resolving mediators (SPMs). SPMs, including resolvins, lipoxinsmaresins, and protectins [16,17], act as ligands for surface receptors [17], particularly the N-formyl peptide receptors (FPRs) family, which can sustain or resolve inflammatory responses depending on the context and SPMs involved. This “promiscuous” family consists of FPR1, FPR2, and FPR3 [18], which have different functional properties [19,20], but are activated by SPMs and trigger various biological functions, including control of pro- or anti-inflammatory reactions [21]. FPRs can sustain inflammatory responses [22], but depending on the context and specific SPMs, they can also play a role in resolving inflammation [23]. The activation of the FPR2 subtype by pro-resolving mediators, known as SPMs, is noteworthy as it plays a role in inflammation resolution [24].

**Figure 1:**
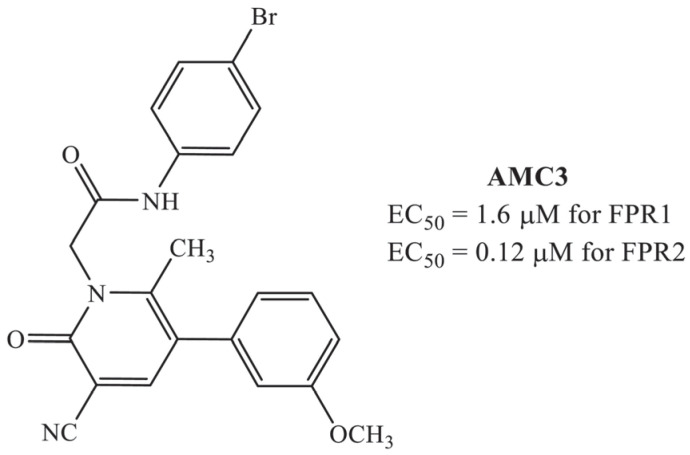
Chemical structure and FPRs activity of AMC3 (**2a** in the original paper).

FPRs have gained attention as potential targets for managing inflammation and pain, particularly in organ-specific or systemic inflammatory diseases like RA [25]. Researchers are exploring newer synthetic pharmaceutical modulators of FPRs [26], including the mixed pyridinone derivative FPR1/FPR2 agonist AMC3 (**2a** in the original paper) (Figure 1) [27]. This compound has shown promising acute pain-relieving activity and efficacy against RA-related hypersensitivity in a rat model [27,28]. The purpose of this new study was to explore the use of AMC3 as a pharmacological modulator of FPRs, deeply investigating its action on primary rat chondrocytes, one of the main cell types involved in this pathology, as well as to study its efficacy, mechanism, and disease-modifying effects against CFA-induced RA.

## 2. Materials and Methods

### 2.1. Chemical Description

Compound **2a** (*N*-(4-bromophenyl)-2-[3-cyano-5-(3-methoxyphenyl)-6-methyl-2-oxopyridin-1(*2H*)-yl]acetamide, AMC3) has been synthesized following the original procedure reported in the corresponding reference [27].

### 2.2. Chondrocytes Isolation and Culture 

Briefly, 4-week-old Sprague–Dawley rats weighing (140 ± 10 g) were sacrificed to collect cartilage from both knees and femoral heads under sterile conditions. Then, the rat cartilage was inserted into a 50 mL tube containing sterile cold phosphate buffered saline (PBS). The cartilage was washed twice with PBS after waiting for the pieces to settle at the bottom of the falcon. The specimens were digested in a 50 mL tube with Dulbecco’s Modified Eagle Medium (DMEM) High Glucose (Merck, Milan, Italy) containing 0.2% collagenase type II from Clostridium histolyticum (Merck, Milan, Italy) for 2–4 h at 37 °C. After digestion, the cellular suspension was centrifuged at 250× *g* for 10 min. The pellet was resuspended, chondrocytes were seeded in 25 cm^2^ cell flasks and raised in DMEM supplemented with 10% fetal bovine serum (FBS; Euroclone, Milan, Italy), 2 mM l-glutamine, 100 U/mL penicillin, and 100 μg/mL streptomycin (Merck, Milan, Italy) at 37 °C in 5% CO_2_ in a cell culture incubator.

### 2.3. 3-(4,5-Dimethylthiazol-2-yl)-2,5-Diphenyltetrazolium Bromide Assay (MTT)

Cell viability was measured using the MTT assay. Chondrocytes were plated at a density of 1.5 × 10^4^ cells/well in 96-well plates (Corning, Tewksbury, MA, USA) and cultured with different concentrations of Interleukin-1β (IL-1β) (Peprotech, Neuilly-sur-Seine, France) (1–30 ng/mL), AMC3 (0.1–100 µM), and with co-treatment of IL-1β (10 ng/mL) and AMC3 (0.1–100 µM). Cell cultures were treated for 24 h at different concentrations, and the percentage of cytotoxicity was measured using the MTT assay. At the end of the treatment, MTT was added to the medium at a final concentration of 1 mg/mL and incubated for 2 h. The supernatants were discarded, and colored formazan crystals were dissolved in 200 µL of dimethyl sulfoxide (DMSO, Merck, Milan, Italy). Absorbance was measured at 550 nm. All results are expressed as % in comparison to the control (arbitrarily set at 100%). Results are expressed as mean ± S.E.M (6 replicates for each experiment and n = 3 experiments). 

### 2.4. Measurement of ROS Production

To evaluate the total amount of intracellular ROS, chondrocytes were seeded in 24-well plates (5 × 10^4^ cells/well; Corning, Tewksbury, MA, USA) and experiments were performed after 24 h. Cells were treated with IL-1β (10 ng/mL) and AMC3 (1–30 µM) for 24 h. After incubation, chondrocytes were washed with PBS and incubated with 10 μg/mL of 2′,7′-dichlorofluorescein diacetate probe (H_2_DCF-DA, Merck, Milan, Italy) for 40 min at 37 °C and 5% CO_2_ and protected from light. Cells were then washed with PBS, detached from the wells with a 0.25% trypsin-EDTA solution, harvested, transferred to a 1.5 mL Eppendorf and centrifuged at 400× *g* for 10 min. The pellet was then broken up and the cells were lysed with a RIPA buffer (50 mM Tris-HCl pH 7,5, 150 mM NaCl, 100 mM NaF, 2 mM EGTA, 1% Triton X-100, 10 μg/mL protease and phosphatase cocktail inhibitors). Cells were centrifuged at 12,000× *g* at 4 °C for 10 min and the supernatants were transferred to a new 0.5 mL Eppendorf and 100 μL of lysate was loaded in duplicate 96-well plates (Corning, Tewksbury, MA, USA). The fluorescence emission was measured at a wavelength of 485/535 nm. Protein concentration was quantified using a bicinchoninic acid (BCA) assay (Merck, Milan, Italy). The ROS production in each sample was normalized to the protein concentration. Control conditions in the absence of treatment were set arbitrarily at 100%. All results are expressed as % compared to the control. The results are expressed as the mean ± S.E.M (3 replicates for each experiment and n = 3 experiments). 

### 2.5. Catalase Activity

Oxidative stress was evaluated by performing a catalase assay. Chondrocytes were plated in 6-well cell culture plates (1.5 × 10^5^ cells/well; Corning, Tewksbury MA, USA) and experiments were performed after 24 h. Cells were treated with IL-1β (10 ng/mL) and AMC3 (1–30 µM) for 24 h. After incubation, the cells were washed once with PBS and scraped off with PBS on ice. The cells were then collected, subjected to a freeze-thaw cycle, and centrifuged at 11,000× *g* for 10 min at 4 °C.

Catalase activity was measured in the supernatant using an Amplex Red Catalase Assay Kit (Invitrogen, Monza, Italy), following the manufacturer’s instructions. Protein concentration was quantified using the BCA assay (Merck, Milan, Italy). Catalase activity in each sample was normalized to protein concentration. Control conditions in the absence of treatment were set arbitrarily at 100%. All results are expressed as % compared to the control. The results are expressed as the mean ± S.E.M (3 replicates for each experiment and n = 3 experiments). 

### 2.6. RNA Isolation, Reverse Transcription and Real Time Polymerase Chain Reaction (RT-PCR) 

Total RNA was isolated from chondrocytes (2 × 10^5^ cells/well; Corning, Tewksbury, MA, USA) and from the thoracic spinal cord using a TRI Reagent (Merck, Milan, Italy). Five hundred nanograms of chondrocyte RNA and one microgram of spinal cord RNA were retrotranscribed using the PrimeScript^TM^ RT reagent kit with a gDNA eraser (Takara Bio, Shinga prefecture, Japan). RT-PCR was performed using SsoAdvanced Universal SYBR^®^ Green Supermix (Bio-Rad, Milan, Italy) according to the thermal profile suggested by the kit. The following rat primers were used: MMP-13: forward: 5′-ATGTGGAGTGCCTGATGTGGGTG-3′, and reverse: 5′-CAGCAGTGCCATCATGGATCCTGG-3′; COX-2: forward: 5′-GGTCTGGTGCCGGGTCTGATGAT-3′, and reverse: 5′-AGTTTGAAGTGGTAACCGCTCAGGTG-3′; ADAMTS-4: forward: 5′-ACCATCAATGGAGATCCGGAGTCGG-3′, and reverse: 5′-CTTGACGTTGCATATGGGACCTCGG-3′; COLIAI: forward: 5′-AGGGTCCCTAATGGTGAGACGTGG-3′, and reverse: 5′-GGTCCCTCGACTCCTATGACTTCTGC-3′; VEGF-A: 5′-AGACTCTTCGAGGAGCACTTTGGGTC-3′, and reverse: 5′-ATGTGTGTGTATGTGGGTGGGTGTGT-3′; 18S: forward: 5′-GGCACCAGACTTGCCCTCCAATG-3′, and reverse: 5′-GGGGAATCAGGGTTCGATTCCG-3′; EAAT1: forward 5′-CAGTCATCGTCGGCCTCCTCATTC-3′, and reverse 5′-CTGGTGATGCGTTTGTCCACACCATTG-3′; S100-β: forward: 5′-TCAGGGAGAGAGGGTGACAAGCAC-3′, and reverse: 5′-GGCTGTGGTCACCATGGAGACGAAG-3′; GFAP: forward: 5′-CTGACACACGTTGTGTTCAAGCAGCC-3′, and reverse: 5′-CTGGAAGGTTAGCAGAGGTGACAAGGG-3′; CCL2: forward: 5′-TCTTCCTCCACCACTATGCAGGTCTC-3′, and reverse: 5′-TCTTTGGGACAGCTGCTGCTGCTGGTG-3′ (Invitrogen, Monza, Italy); validated primers for rNos2 and rEAAT2, and rActb and rGAPDH were purchased from Bio-Rad (qRnoCID0017722, qRnoCED0005967, qRnoCID0056984 and qRnoCID0057018). The differential expression of transcripts was normalized on the housekeeping gene expression level. 

### 2.7. Animals

Male Sprague–Dawley rats (Envigo, Varese, Italy) weighing approximately 200–250 g at the beginning of the experimental procedure were used. Animals were housed in Ce.S.A.L. (Centro Stabulazione Animali da Laboratorio, University of Florence) and used at least one week after their arrival. Four rats were housed per cage (size 26 × 41 cm^2^); animals were fed a standard laboratory diet and tap water ad libitum, kept at 23 ± 1 °C with a 12 h light/dark cycle, light at 7 a.m. All animal manipulations were carried out according to Directive 2010/63/EU of the European Parliament and of the European Union Council (22 September 2010) on the protection of animals used for scientific purposes. The ethical policy of the University of Florence complies with the Guide for the Care and Use of Laboratory Animals of the US National Institutes of Health (NIH Publication No. 85-23, revised 1996; University of Florence Assurance Number: A5278-01). Formal approval to conduct the experiments was obtained from the Italian Ministry of Health (No. 517/2017, 06/04/2017) and the Animal Subject Review Board of the University of Florence. Animal experimentation has been reported according to the ARRIVE guidelines (McGrath and Lilley, 2015). All efforts were made to minimize animal suffering and to reduce the number of animals used.

### 2.8. Complete Freund’s Adjuvant-Induced Inflammatory Arthritis

Articular damage was induced by an injection of Complete Freund’s Adjuvant (CFA) (Merck, Milan, Italy) into the tibio–tarsal joint [29,30]. Briefly, rats were lightly anesthetized with 2% isoflurane. After sterilizing the skin of the left leg with 75% ethyl alcohol, the lateral malleolus was located by palpation, and a 28-gauge needle was inserted vertically to penetrate the skin and turned distally for insertion into the articular cavity at the gap between the tibiofibular and tarsal bone until a distinct loss of resistance was felt. A volume of 50 μL of CFA was then injected (left paw, ipsilateral). Control rats received 50 μL of saline solution in the tibio–tarsal joint. Behavioral tests were performed 7 and 14 days after the CFA injection.

### 2.9. FPR agonist Administration

AMC3 (10 mg kg^−1^) was suspended in a 1% carboxymethylcellulose sodium salt solution (CMC) and administered per os (p.o.) beginning on the day of intra-articular (i.a.) injection of CFA. Behavioral measurements were conducted on days 7 and 14. Dosage was established in previous experiment [28].

### 2.10. Paw Pressure Test

The nociceptive threshold was determined with an analgesimeter (Ugo Basile, Varese, Italy). Briefly, constantly increasing pressure was applied to a small area of the dorsal surface of the hind paw using a blunt conical mechanical probe. Mechanical pressure was increased until vocalization or withdrawal reflex occurred, while the rats were lightly restrained. Vocalization or withdrawal reflex thresholds are expressed in grams. In this study, an arbitrary cut-off value of 100 g was used. Data were collected by an observer who was blinded to the protocol [31].

### 2.11. Incapacitance Test

Weight bearing changes were measured using an Incapacitance apparatus (Linton Instrumentation, Norfolk, UK) detecting changes in postural equilibrium after a hind limb injury. As described in Di Cesare Mannelli L et al., 2016 [32], rats were trained to stand on their hind paws in a box with an inclined plane (65° from horizontal). This box was placed above the Incapacitance apparatus. This allowed us to independently measure the weight that the animal applied on each hind limb. The mean of five consecutive measurements is reported for each animal. In the absence of a hindlimb injury, rats applied equal weight to both hind limbs, indicating postural equilibrium, whereas an unequal distribution of weight on the hind limbs indicated a monolateral decrease in pain threshold. Data are expressed as the difference between the weight applied to the limb contralateral to the injury and the weight applied to the ipsilateral limb (Δ weight).

### 2.12. Histological Evaluation

Animals were sacrificed by cervical dislocation. The legs were cut under the knees, flayed, and fixed in 4% formaldehyde in PBS for 48 h at room temperature. Subsequently, samples were decalcified by treatment with 0.76 M sodium formate, 1.6 M formic acid solution in H_2_O for 4 weeks with a change of solution every 7 days. At the end of the decalcification, the samples were dehydrated in alcohol and embedded in paraffin. Sections (6 µm thick) of the tibio–tarsal joint were stained with hematoxylin and eosin and qualitatively analyzed by two independent observers in a blinded fashion. Several morphological parameters (inflammatory infiltrate, fibrosis, bone, and cartilage erosion) were observed and quantified by a specific score (0: absent; 1: light; 2: moderate; 3: severe) according to Snekhalatha and colleagues [33].

### 2.13. Immunofluorescence Analysis 

After animal sacrifice, the L4–L5 segments of the spinal cord were exposed from the lumbovertebral column via laminectomy and identified by tracing the dorsal roots from their respective dorsal root ganglion. Formalin-fixed cryostat sections (7 μm) were washed with 3× PBS and 0.3% Triton X-100 for 5 min, and then incubated at room temperature, for 1 h in blocking solution (PBS, 0.3% Triton X-100, and 5% albumin bovine serum; PBST). Slices were incubated overnight at 4 °C with primary antibodies directed against glial fibrillary acidic protein (GFAP; rabbit, 1:500; DAKO, Carpinteria, CA, USA) for astrocyte staining. The following day, the slides were washed with 3× PBS and 0.3% Triton X-100 for 5 min and then incubated in goat anti-rabbit IgG secondary antibody labeled with Alexa Fluor 568 (1:500; Invitrogen, Carlsbad, CA, USA) at room temperature for 2 h. After washing with 3× PBS with 0.3% Triton X-100 for 10 min, the sections were incubated with DAPI, a nuclear marker, at room temperature for 5 min, and the slides were mounted using Fluoromount™ (Life Technologies-Thermo Scientific, Rockford, IL, USA). Negative control sections (no exposure to primary antisera) were processed simultaneously with the other sections for all immunohistochemical studies to exclude the presence of nonspecific immunofluorescent staining or cross immunostaining. Images were acquired using a motorized Leica DM6000B microscope equipped with a DFC 350 FX camera (Leica, Mannheim, Germany).

The mean fluorescence intensity of vascular endothelial growth factor-A (VEGF-A) in the control and oxaliplatin-treated animals was calculated by subtracting the background (multiplied by the total area) from the VEGF-A integrated intensity. Analyses were performed on 3 different images for each animal, collected using a 20X objective. GFAP-positive cells were counted using the “cell counter” plugin of ImageJ (NIH, Bethesda, MD, USA).

### 2.14. Statistical Analysis 

Results were expressed as mean ± S.E.M. and analysis of variance was performed using ONE-way ANOVA. Bonferroni’s significant difference procedure was used for post-hoc comparisons. P-values less than 0.05 were considered significant; * *p* < 0.05; ** *p* < 0.01; *** *p* < 0.001 vs. control, and ^ *p* < 0.05; ^^ *p* < 0.01; ^^^ *p* < 0.001 vs. treatment. The data were analyzed using ‘Origin 9.1’.

## 3. Results

### 3.1. RA Modelling In Vitro and Evaluation of AMC3 Effects on Primary Rat Chondrocytes Viability 

**Figure 2:**
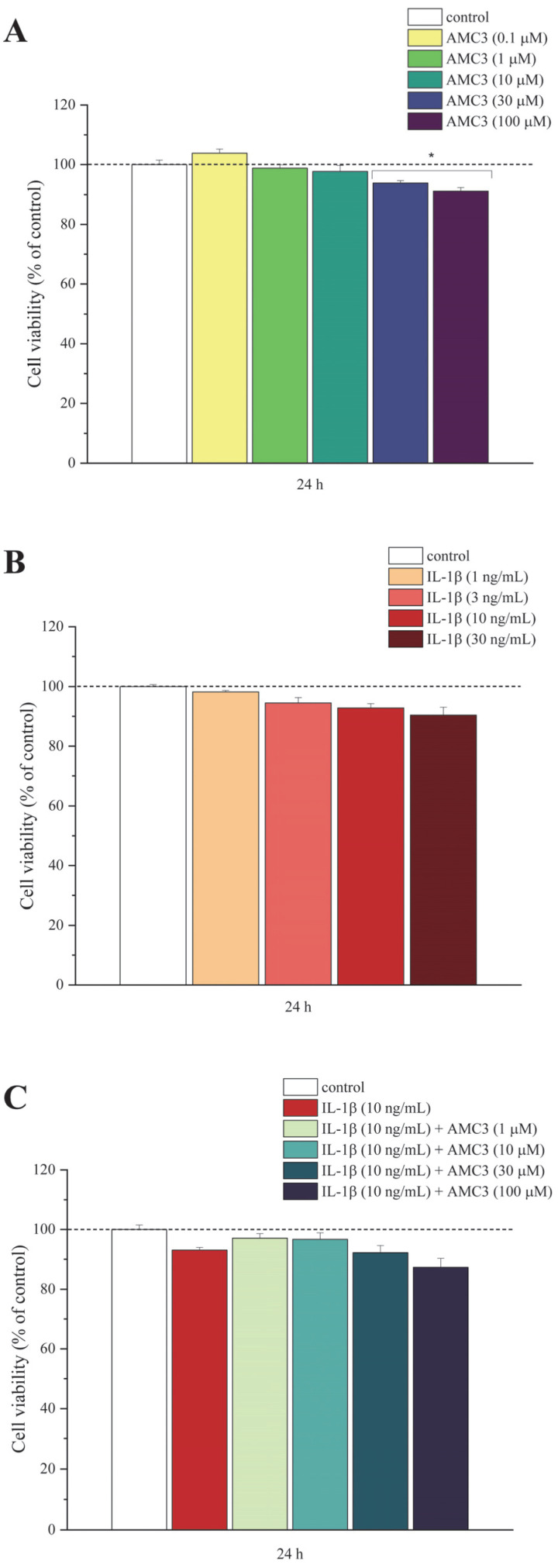
Effects of AMC3 and/or IL-1β on the viability of primary rat chondrocytes. Chondrocytes were exposed to (**A**) AMC3 and (**B**) IL-1β for 24 h at concentrations ranging from 0.1 to 100 µM and 1 to 30 ng/mL, respectively. (**C**) Chondrocytes were treated with AMC3 (0.1–100 µM) in combination with IL-1β (10 ng/mL) for 24 h. All results are expressed as percentages compared to the control (arbitrarily set at 100%). Results are expressed as mean ± S.E.M, and analysis of variance was performed using ONE-way ANOVA. Bonferroni’s significant difference procedure was used for post-hoc comparisons. * *p* < 0.05 vs. control. The data were analyzed using the Origin 9.1 software.

The hypothetical cytotoxic effect of AMC3 was evaluated on primary normal chondrocytes using the MTT assay. Chondrocyte cells were treated for 24 h with increasing concentrations of AMC3 (0.1–100 µM), and a slight reduction in cell viability (by about 10–15%) emerged only at the highest AMC3 concentrations (30–100 µM), as shown in Figure 2A. In order to model RA in vitro, chondrocytes were stimulated with IL-1β, and firstly, the effect of treatment with different concentrations of IL-1β (1–30 ng/mL) on cell viability was evaluated. After 24 h, none of the IL-1β concentrations tested particularly affected cellular viability (Figure 2B) and so, also based on literature [34], 10 ng/mL was chosen as the best concentration to induce chondrocytic alterations, the optimal condition to test AMC3 efficacy to rescue several parameters, like oxidative stress and changes in genes expression profile, as reported below. Additionally, the evaluation of co-treatment for 24 h with IL-1β (10 ng/mL) and AMC3 (0.1–100 µM) showed no significant toxicity in chondrocytes (Figure 2C), confirming the non-toxic effect of this novel FPRs agonist and allowing the selection of 1 and 30 µM as concentrations to be further tested.

### 3.2. AMC3 Counteracts IL-1β-Exacerbated Oxidative Stress 

**Figure 3:**
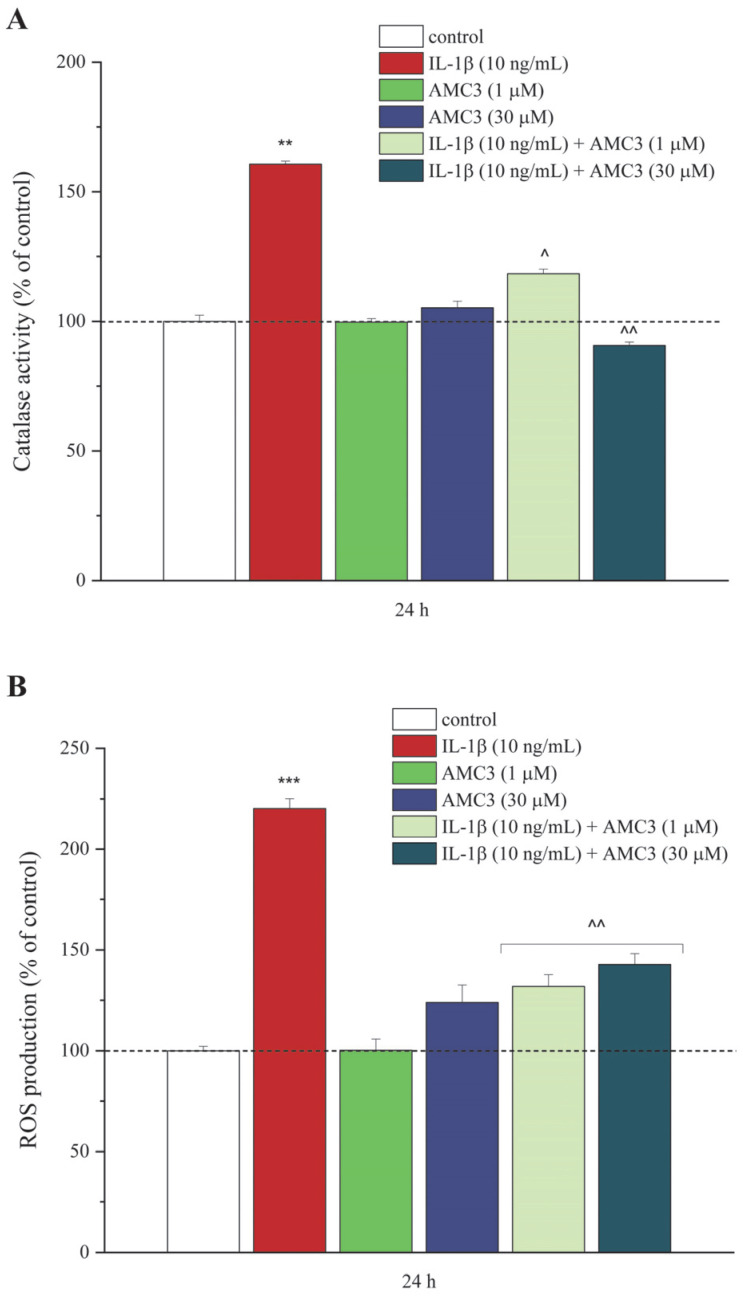
Effects of AMC3 on IL-1β-induced oxidative stress in primary rat chondrocytes. Increased oxidative stress levels in primary rat chondrocytes induced by IL-1β (10 ng/mL) were significantly reduced after 24 h of co-treatment with AMC3 (1–30 µM), as indicated by (**A**) catalase enzyme activity and (**B**) intracellular ROS production levels. All results are expressed as percentages compared to the control (arbitrarily set at 100%). Results were expressed as mean ± S.E.M, and analysis of variance was performed using ONE-way ANOVA. Bonferroni’s significant difference procedure was used for post-hoc comparisons. ** *p* < 0.01 and *** *p* < 0.001 vs. control; ^ *p* < 0.05 and ^^ *p* < 0.01 vs. IL-1β (10 ng/mL). The data were analyzed using the Origin 9.1 software.

The antioxidant effects of AMC3 (1–30 µM) after 24 h of treatment on oxidative imbalance in IL-1β-stimulated chondrocytes showed a concentration-dependent decrease on enzymatic activity of catalase in AMC3 co-treated samples, compared to IL-1β alone, respectively, about 40 and 70% (Figure 3A). The single treatment with AMC3 (1–30 µM) was unable to influence the activity of this enzyme. 

Furthermore, we examined the positive action of AMC3 against redox unbalance in the same experimental setting through the evaluation of intracellular ROS production using an H_2_DCF-DA probe that, once spread into the cells, was oxidized by ROS into a highly fluorescent compound—2′, 7′-dichlorophluorescein (DCF). Briefly, as showed in Figure 3B, 24 h of the AMC3 treatment (1–30 µM) significantly prevented the twofold increase of H_2_DCF-DA fluorescence induced by IL-1β (10 ng/mL), meanwhile, AMC3 per se did not significantly modify the ROS levels production.

### 3.3. AMC3 Modulates Chondrocytic Expression Genes Involved in Inflammation and Tissue Structural Integrity Maintenance 

**Figure 4:**
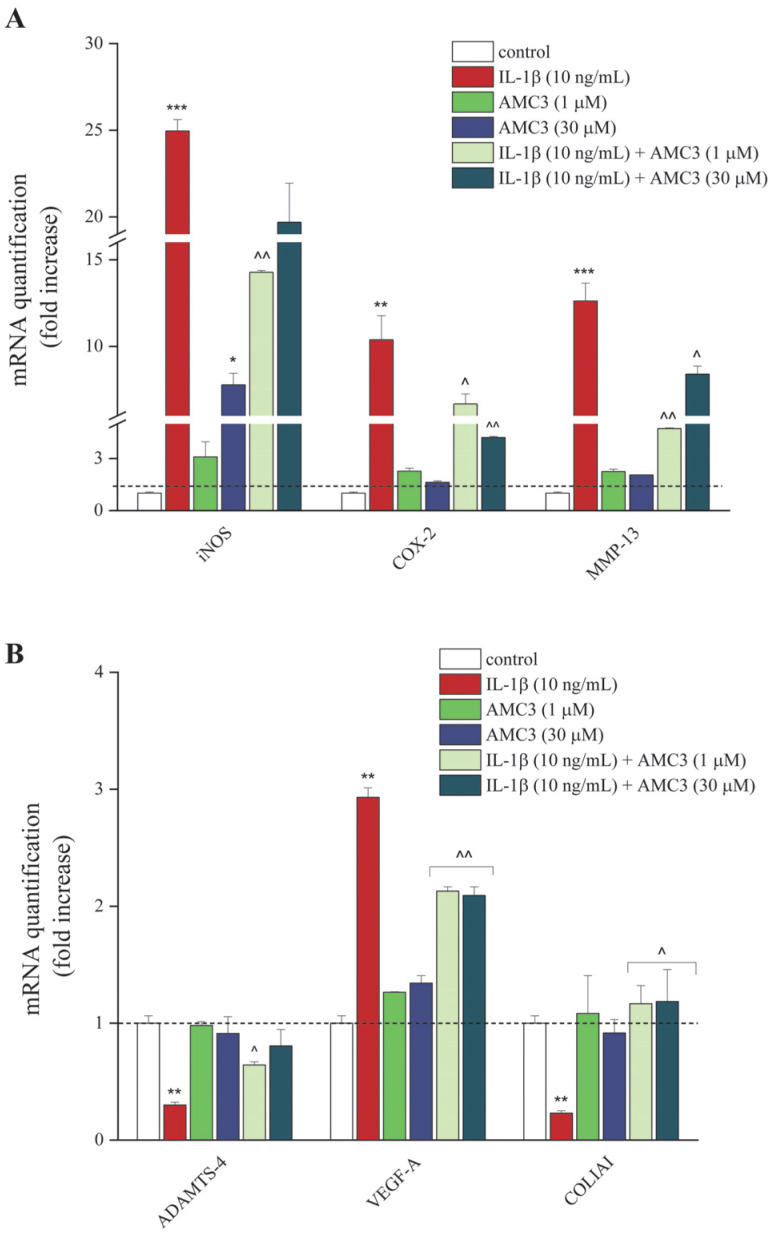
AMC3 (1–30 µM) regulates the expression of pro-inflammatory genes and mediators involved in cartilage protection and remodeling. (**A**) The levels of mRNA expression of pro-inflammatory mediators and (**B**) resolution mediators were measured by RT-PCR in chondrocytes after treatment for 24 h with IL-1β alone (10 ng/mL), and in co-treatment with AMC3 (1–30 µM). The control was arbitrarily set at 1 and the mRNA levels were expressed as mean ± SEM (normalized on the expression of Actin, chosen as housekeeping gene). * *p* < 0.05; ** *p* < 0.01 and *** *p* < 0.001 vs. control; ^ *p* < 0.05 and ^^ *p* < 0.01 vs. IL-1β (10 ng/mL).

Through RT-PCR analysis, we evaluated changes in the mRNA expression levels of inducible nitric oxide synthase (iNOS), cyclooxygenase-2 (COX-2), MMP-13, ADAMTS-4, VEGF-A, and collagen type I alpha 1 chain (COLIAI)—some genes involved in inflammatory processes and in the maintenance of tissue structural integrity—in untreated chondrocytes (control), as well as in cells treated with IL-1β (10 ng/mL) and/or AMC3 (1–30 µM) for 24 h. We observed, as reported in Figure 4A,B, that AMC3 (1–30 µM) alone did not change the gene expression profile of chondrocytes, except for iNOS levels in the 30 µM condition, that increased sevenfold. On the contrary, all the investigated genes were up-regulated by IL-1β (to be noted the twenty-fivefold increase of iNOS and the twelvefold increase of COX-2 and MMP-13), except for COLIAI that was down-modulated approximately four times compared to the control.

Meanwhile, both concentration of AMC3 (1–30 µM) significantly prevented the enhancement of iNOS, COX-2, MMP13, VEGF-A, and ADAMTS-4 genes by about 30–70%, as well as promoted a marked overexpression of the COLIAI (fivefold increase), compared to the IL-1β condition. 

### 3.4. Pain-Relieving Effect of AMC3 on CFA-Induced Rheumatoid Arthritis 

**Figure 5:**
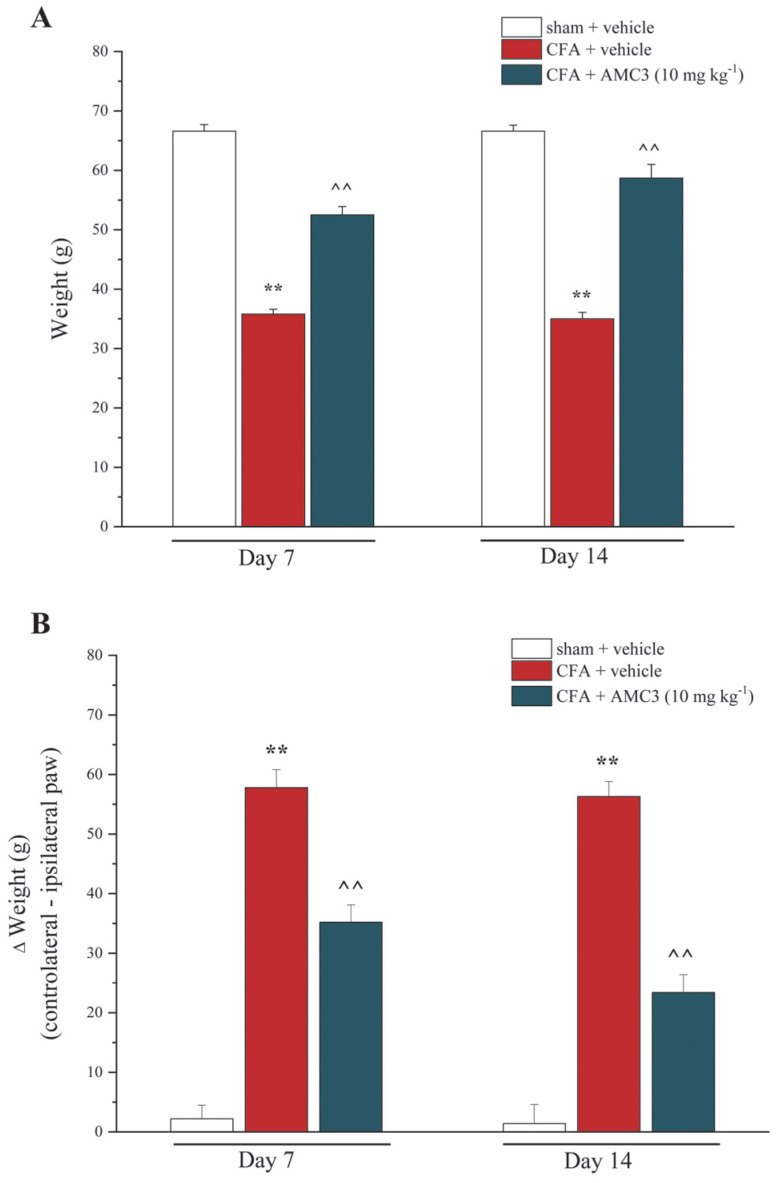
Effect of repeated treatment with AMC3 at 10 mg kg^−1^. (**A**) The Paw Pressure test was performed to evaluate hypersensitivity to noxious mechanical stimuli. (**B**) The Incapacitance test measures hind limb weight-bearing alterations as postural imbalance related to pain. Data are expressed as the difference between the weight applied to the limb contralateral to the injury and the weight applied to the ipsilateral limb (Δ weight). Measurements were performed on days 7 and 14 after CFA i.a. injection. AMC3 was suspended in 1% CMC and administered daily. The control animals were treated with the vehicle. The values represent the mean of six rats in two different experimental sets. ** *p* < 0.01 vs. vehicle + vehicle–treated-animals; ^^ *p* < 0.01 vs. CFA-treated animals.

The beneficial effect of AMC3 (10 mg kg^−1^) in chronic treatment (14 days) was evaluated in an in vivo model of RA. Rats were treated intra-articularly (left paw, ipsilateral) with CFA on day 1. AMC3 was daily per os. administered at the dose of 10 mg kg^−1^, starting from the same day of CFA intra-articular injection. The dose was chosen based on previous experiments [28]. Seven and Fourteen days after damage, the mechanical withdrawal threshold to a noxious stimulus (Paw Pressure test, Figure 5A) and spontaneous pain (Incapacitance test, Figure 5B) were measured. 

As shown by the Paw Pressure test, AMC3 (10 mg kg^−^^1^) treatment significantly increased the withdrawal threshold in response to a noxious mechanical stimulus, in comparison to CFA + vehicle animals, in which the ipsilateral paw pain threshold was particularly low, starting from day 7 (52.5 ± 1.4 g for CFA + AMC3 vs. 35.8 ± 0.8 g for CFA) until day 14 (58.7 ± 2.3 g for CFA+ AMC3 vs. 35 ± 1.1 g for CFA). Similarly, this compound was also able to improve in a statistically significant manner the postural imbalance caused by CFA-injection both 7 (37.2 ± 2.9 g for CFA + AMC3 vs. 57.8 ± 3 g for CFA) and 14 (23.4 ± 3 g for CFA+ AMC3 vs. 56.3 ± 2.5 g for CFA) days after the damage (Figure 5B).

### 3.5. AMC3 Improves Morphological Alterations of Tibio-Tarsal Joint

**Figure 6:**
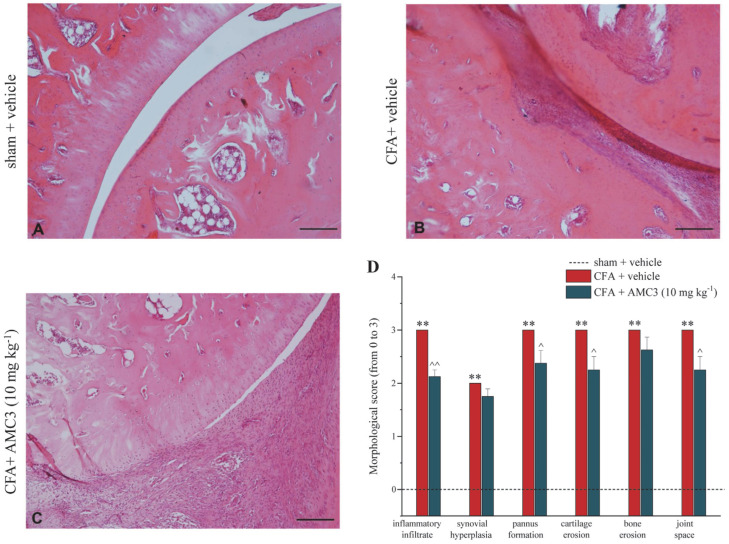
Effects of AMC3 on morphological derangement of tibio–tarsal joint. Joints were collected on day 14 after repeated treatment with AMC3 (10 mg kg^−1^, daily from day of CFA injection). Sections (6 μm) of paraffin-embedded joints were analyzed by hematoxylin and eosin staining. Comparative images of the tibio–tarsal joint of (**A**) control animals (vehicle + vehicle), (**B**) CFA + vehicle, and (**C**) CFA + AMC3 (10 mg kg^−1^). Panel D shows the scores of morphological alterations (from 0 to 3). Pictures are representative of histological preparations from six rats for each group (in two different experimental sets) and two sections for each animal were analyzed. ** *p* < 0.01 vs. sham + vehicle–treated-animals; ^ *p* < 0.05 and ^^ *p* < 0.01 vs. CFA-treated animals. Scale bar 100 µm.

The study sought to understand the effect of chronic administration of AMC3 (10 mg kg^−1^) on the deterioration of the tibio–tarsal joint 14 days after an injection of CFA in comparison to control group animals (Figure 6A).

The morphological evaluation results reported in Figure 6C highlighted that AMC3 significantly reduced the inflammatory infiltrate that, in CFA-treated animals, is present in the fibrous tissue, up to completely replacing the joint space (2.12 ± 0.125 for CFA + AMC3 10 mg kg^−1^ vs. 3 ± 0.1 for CFA), as well as the pannus formation (Figure 6B). Moreover, it is worth noting the reduction mediated by the action of chronic-administered AMC3 (10 mg kg^−1^) on cartilage and bone erosion foci, as well as on the preservation of joint space (Figure 6C,D).

### 3.6. AMC3-Modulated Expression of Inflammatory and Painful-Related Genes in Spinal Cord

AMC3 protective mechanisms were explored also looking at changes in gene expression profiles of pro-inflammatory cytokines and pain-related factors in nervous tissue, particularly in the spinal cord.

**Figure 7:**
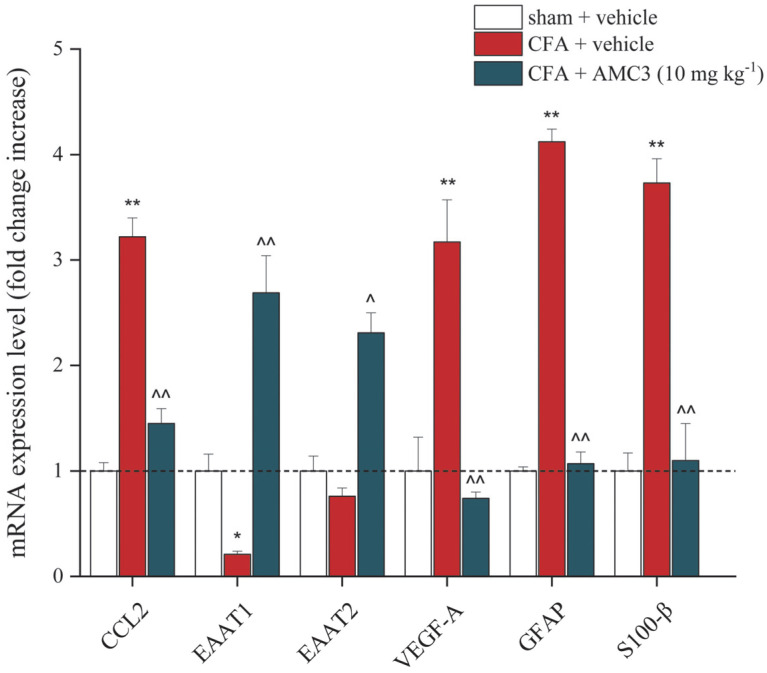
Effects of AMC3 on inflammatory and pain-related gene expression. Relative gene expression of CCL2, EAAT1, EAAT2, VEGF-A, GFAP, and S100-β in the spinal cord was measured by RT-PCR on day 14 after the CFA injection. The control was arbitrarily set at 1, and the mRNA levels were expressed as the mean ± S.E.M (normalized to the expression of GAPDH, chosen as the housekeeping gene). * *p* < 0.05 and ** *p* < 0.01 vs. vehicle  +  vehicle; ^ *p* < 0.05 and ^^ *p* < 0.01 vs. CFA + vehicle.

Figure 7 highlighted the ability of AMC3 (10 mg kg^−1^) to reduce the CFA-mediated threefold increase of CCL2 and VEGF-A, 2 critical factors involved in neuropathic pain. In addition, AMC3 (10 mg kg^−1^) restored the expression of the astrocytic Excitatory Amino Acid Transporter 1 (EAAT1) involved in glutamate re-uptake, which was strongly compromised by CFA treatment (Figure 7). 

Finally, we found that AMC3 (10 mg kg^−1^) mitigated the CFA-induced astrogliosis, as shown by the fourfold decrease of mRNA expression levels of GFAP and S100 calcium-binding protein β (S100-β), which was further confirmed by immunofluorescence analysis.

### 3.7. AMC3 Impairs Astroglial Activation in Spinal Cord

**Figure 8:**
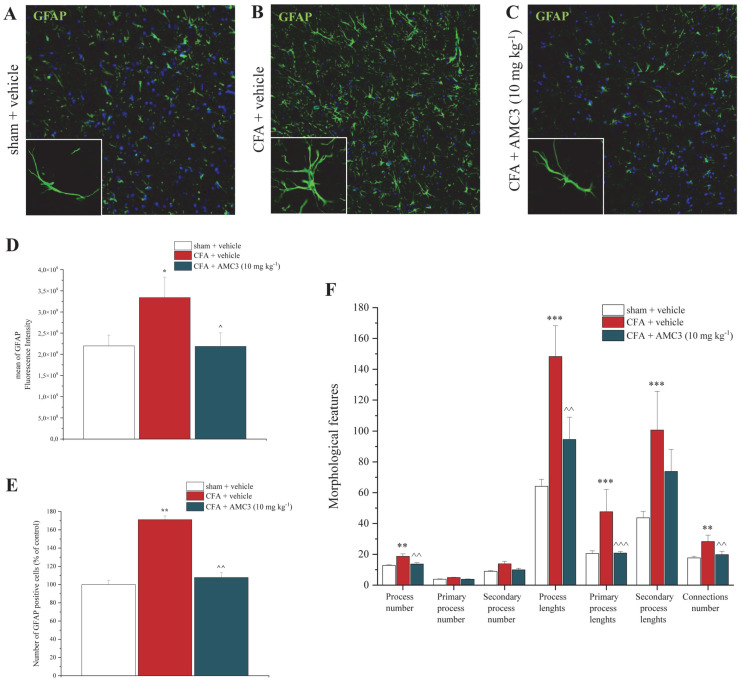
Effects of AMC3 (10 mg kg^−1^) on CFA-dependent astrogliosis in the spinal cord. On day 14, fluorescence intensity (**D**), number of GFAP+ cells (**E**), and morphological features (**F**) were measured in the dorsal horn of the spinal cord. Representative immunohistochemical staining of (**A**) control animals (sham + vehicle), (**B**) CFA + vehicle, and (**C**) CFA + AMC3 (10 mg kg^−1^). Original magnification 20X for all images and 63X for details in the box. Each value represents the mean  ±  S.E.M of six rats per group (performed in two different experimental sets). * *p* < 0.05, ** *p* < 0.01 and *** *p* <  0.001 vs. sham  +  vehicle; ^ *p* < 0.05, ^^ *p* < 0.01 and ^^^ *p* < 0.001 vs. CFA + vehicle.

In line with the RT-PCR findings, we discovered that the repeated administration of the FPRs modulator, AMC3 (10 mg kg^−1^), was able to counteract astrogliosis on nervous tissue collected 14 days after the CFA injection and AMC3 administration. Figure 8C shows that the chronic treatment with AMC3 (10 mg kg^−1^) promoted the mitigation of the spinal astrocytes hyperactivation induced by the CFA injection (Figure 8B,C) compared to control group animals (Figure 8A), established as a decrease of 50% of fluorescence intensity of GFAP (Figure 8D) and a reduction of 60% in the number of GFAP^+^ cells (Figure 8E) in comparison to the CFA condition (Figure 8B–E). 

Furthermore, from the morphometric analysis emerged a reduction of about 30% in the number of total astrocyte processes, as well as a 70% reduction in total length and about 40% of a connections number, in animals treated with AMC3 compared to the CFA + vehicle group, indicating a reduced cell activation and an astrogliosis mitigation (Figure 8F).

## 4. Discussion

The management of rheumatoid arthritis, a long-term autoimmune disorder characterized by a relevant inflammatory state due to the production of abnormal quantities of autoantibodies [35], an imbalance between the pro- and anti-inflammatory activities of cytokines [36], and a synovial hyperplasia that leads to structural changes and gradually joint wrecking [37], is the common goal that has always bedeviled researchers and clinicians. 

Commonly used pharmacological interventions for RA aim to promptly limit persistent inflammation in order to ease the symptoms and slow down disease progression [38]. However, in light of the evidences that (i) less than 50% of treated patients are destined to reduce or, more rarely, complete remission of the disease [39]; (ii) specialized RA medications are much more expensive [4]; and (iii) pharmacological treatments, such as oral NSAIDs, GCs, and DMARDs, are often associated with serious adverse effects [40], the development of novel molecules become necessary to counteract the onset of characteristic chronic inflammation and to avoid irreversible joint or organ damage [39,41].

The present results described the pharmacological activity of AMC3, a synthetic modulator of FPRs with a pyridinone scaffold and a higher affinity for FPR2 (EC_50_ = 1.6 µM for FPR1 and 0.12 µM for FPR2) [27]. Preliminarily reported as a pain reliever compound [28], here, it emerges, in vitro and in vivo, as a valuable compound to counteract RA deeply affecting pathological mechanisms. 

Firstly, we modelled RA in vitro through stimulation of isolated primary rat chondrocytes with IL-1β—one of the key driving molecules in the majority of RA animal models [42,43]—in order to modify chondrocytes’ behavior and metabolism [44] and to promote oxidative stress [45,46], an event that contributes importantly to the pathogenesis of several human diseases, including RA [43,47]. Our results demonstrated, firstly, a good in vitro effect of the FPRs modulator, AMC3, on chondrocytes’ viability, and also its important antioxidant properties and chondroprotective effects. In particular, in this study emerged the ability of AMC3 to restore, in a concentration-dependent manner, the enzymatic dysfunction of catalase—a key enzyme of the antioxidant machinery [48,49]—as well as to block IL-1β-induced oxidative stress that can lead to the disruption of normal redox signaling and cellular homeostasis [50,51]. These findings are in agreement with results obtained by Martinez and colleagues that highlighted the effects of BML-111—a commercial synthetic ALX/FPR2 receptor agonist—on UVB-induced oxidative stress [52]. 

Oxidative stress and ROS also activate several signaling pathways and play a major role in the pathophysiology of RA, particularly on the interaction between iNOS and COX-2 systems that induces apoptosis in chondrocytes as well as regulates the ECM degradation by increasing the activation of MMPs [53]. Our findings highlighted the beneficial properties of AMC3 on changes in the RA chondrocytic gene expression profile induced by pro-inflammatory IL-1β. Especially since its inhibitory effects are emerged through an iNOS overproduction, an enzyme primarily linked with the chronic and progressive inflammation [54]; on COX-2, a remarkable enzyme involved in prostaglandin biosynthesis—in particular, that of Prostaglandin E_2_ (PGE_2_), which is responsible for a matrix metalloproteinase unbalance [55]; and an increase in the expression of the angiogenic factor VEGF-A [56], that contributes to inflammatory hyperalgesia [57,58], effects potentially promoted by the suppression of NF-κB activity [59]; as well as of the influence exerted by the activation of these receptors on MAPK pathways, including ERK, p38 MAPK, and JNK [60]. The present results are consistent with the data reported in the literature about the FPR2-mediated anti-inflammatory and pain-relieving effects of two endogenously generated agonists, like Annexin1 (ANXA1) [61,62] and lipoxins (LX) A4 [63]. On a similar vein, the novel pyridinone derivative drug, AMC3, strongly attenuated the actions of MMP and ADAMTs species on cartilage destruction, a phenomenon increased by the ROS-mediated damage to the mitochondrial DNA [64]—particularly of MMP-13, one of the principal proteolytic enzymes that degrade interstitial collagens, including collagen types I, II, and III [65,66,67], and of ADAMTS-4, a master regulator of cartilage proteoglycan (aggrecan) degradation in arthritic disease [68]. Moreover, AMC3-mediated significant restoration of normal mRNA expression levels of COLIAI, strongly compromised by IL-1β stimulation, confirmed the beneficial effects of this modulator to reduce the inflammatory and degradative processes in the chondrocytes. 

Additionally, another important finding aroused in our research is the AMC3 modulation of angiogenesis-related mediators’ synthesis, RA-related phenomenon particularly sustained during the onset of the disease, like VEGF-A [22,44,51,53]. Angiogenic activity amplifies inflammation, joint damage, and pain [69,70,71,72,73]. In recent years, accumulating evidence has suggested the pathological involvement of VEGF-A and its signaling pathways in the disease progression and associated pain in various rheumatic diseases, including RA, applying this growth factor as a hallmark of chronic pain and intensifying the study of VEGF-A and its cognate receptors, VEGFR-1 and VEGFR-2, as a therapeutic target for pain treatment [57,74].

In vitro efficacy was confirmed by in vivo studies on the CFA model, characterized by an increasing pain sensitivity starting from 3 days after injection [75], and by a great similarity with the human RA [76,77]. The bi-weekly treatment with AMC3 has induced protective and pain-killer effects by promoting a significant restoration of the pain threshold to noxious mechanical stimulus, as well as to spontaneous pain caused by CFA. The demonstrated efficacy over a two-week treatment allows us to exclude tolerance development; furthermore, since the pain-relieving properties were improved over time and clearly evidenced 24 h after treatment, a disease-modifying effect of AMC3 is suggested. For this reason, we studied, ex vivo, the histological and molecular state of the joint as the primary target of RA, and the central nervous system since it has a pivotal role in pain chronicization [27,28]. Histology revealed that AMC3 facilitated morphological recovery, showing a significant improvement in cartilage architecture, highlighting a reduction in the volume of the paw, better arthritic scores, and a general improvement in histopathology of articular cartilage by reducing inflammation and the formation of invading pannus, which is generally promoted by CFA injection [10].

Accumulating evidence suggests that, beyond peripheral inflammation, structural and neurochemical changes within the joint and sensory nervous system are implicated in the association between RA pain, tissue injury, and inflammatory processes in the joints [78]. Glial cells, especially astrocytes, are particularly activated in response to peripheral inflammation and can contribute to the maintenance of chronic pain by releasing neuromodulators, such as growth factors (VEGF-A), proinflammatory cytokines (iNOS, COX-2, MMP-13, ADAMTS-4), and chemokine (CCL2), and modifying glutamate metabolism by downregulating its transporters [79,80]. In our research, AMC3 induced antinociceptive effects after repeated administration in CFA-induced inflammatory pain, prevented CFA-induced overproduction of CCL2 and VEGF-A, and restored the correct expression of glutamate transporters, especially EAAT1 and EAAT2, which are strongly compromised by CFA [81]. Furthermore, this FPR agonist decreased the CFA-induced upregulation of GFAP and S-100β mRNA expression, as well as reduced astrogliosis by decreasing the fluorescence intensity of GFAP increased by CFA and all typical features of increased astrocytic reactivity, such as augmenting cell volume, hypertrophy and thickening/retraction of branches and networking [62]. The implications of these findings are that the pain-reducing effects of AMC3 at the spinal cord level may be due, in part, to the reduced cytokine production from astrocytes that results from prolonged exposure to nociceptive stimuli.

## 5. Conclusions

Although it is important to recognize that no gender studies have been conducted on the efficacy of AMC3—making further future investigations in females necessary, which will further enrich our knowledge on the subject—to date, it has been possible to highlight the protective antioxidant and anti-inflammatory effects of AMC3 in chondrocytes, as well as its pain-relieving and disease-modifying properties in male RA rats. Protective effects on both joints and the nervous system have been shown. The FPRs agonist AMC3 emerges as a candidate for a long-lasting, disease-modifying treatment of RA. Protective effects, both in joints and the nervous system, were highlighted. The FPRs agonist AMC3 emerges as a candidate for a long lasting, disease modifying treatment of RA.

## Data Availability

The data presented in this study are available on request from the corresponding author.
